# Promoting fairness in link prediction with graph enhancement

**DOI:** 10.3389/fdata.2024.1489306

**Published:** 2024-10-24

**Authors:** Yezi Liu, Hanning Chen, Mohsen Imani

**Affiliations:** ^1^Department of Electrical Engineering and Computer Science, University of California, Irvine, Irvine, CA, United States; ^2^Donald Bren School of Information and Computer Sciences, University of California, Irvine, Irvine, CA, United States

**Keywords:** fairness, large-scale graphs, link prediction, trustworthy graph neural network, data-centric machine learning

## Abstract

Link prediction is a crucial task in network analysis, but it has been shown to be prone to biased predictions, particularly when links are unfairly predicted between nodes from different sensitive groups. In this paper, we study the fair link prediction problem, which aims to ensure that the predicted link probability is independent of the sensitive attributes of the connected nodes. Existing methods typically incorporate debiasing techniques within graph embeddings to mitigate this issue. However, training on large real-world graphs is already challenging, and adding fairness constraints can further complicate the process. To overcome this challenge, we propose FairLink, a method that learns a fairness-enhanced graph to bypass the need for debiasing during the link predictor's training. FairLink maintains link prediction accuracy by ensuring that the enhanced graph follows a training trajectory similar to that of the original input graph. Meanwhile, it enhances fairness by minimizing the absolute difference in link probabilities between node pairs within the same sensitive group and those between node pairs from different sensitive groups. Our extensive experiments on multiple large-scale graphs demonstrate that FairLink not only promotes fairness but also often achieves link prediction accuracy comparable to baseline methods. Most importantly, the enhanced graph exhibits strong generalizability across different GNN architectures. FairLink is highly scalable, making it suitable for deployment in real-world large-scale graphs, where maintaining both fairness and accuracy is critical.

## 1 Introduction

The scale of graph-structured data has expanded rapidly across various disciplines, including social networks (Liben-Nowell and Kleinberg, 2003), citation networks (Yang et al., 2016), knowledge graphs (Liu et al., 2023; Zhang et al., 2022), and telecommunication networks (Nanavati et al., 2006; Xie et al., 2022). This growth has spurred the development of advanced computational techniques aimed at modeling, discovering, and extracting complex structural patterns hidden within large graph datasets. Consequently, research has increasingly focused on inference learning to identify potential connections, leading to the creation of algorithms that enhance the accuracy of link prediction (Mara et al., 2020; Li et al., 2024). Despite the strong performance of these models in link prediction, they can exhibit biases in their predictions (Angwin et al., 2022; Bose and Hamilton, 2019). These biases may result in harmful social impacts on historically disadvantaged and underserved communities, particularly in areas such as ranking (Karimi et al., 2018), social perception (Lee et al., 2019), and job promotion (Clifton et al., 2019). Given the widespread application of these models, it is crucial to address the fairness issues in link prediction.

Many existing studies have introduced the concept of fairness in link prediction and proposed algorithms to achieve it. For instance, FairAdj (Li et al., 2021) introduces *dyadic fairness*, which requires equal treatment in the prediction of links between two nodes from different sensitive groups, as well as between two nodes from the same sensitive group. These approaches are predominantly model-centric, incorporating debiasing methods during the training process (Rahman et al., 2019; Masrour et al., 2020; Tsioutsiouliklis et al., 2021; Li et al., 2021; Current et al., 2022). However, promoting fairness in models trained on large-scale graphs is particularly challenging. State-of-the-art link predictors, often deep learning methods like GNNs, are already difficult to train on large graphs (Zhang S. et al., 2021; Hu et al., 2021; Ferludin et al., 2022; Han et al., 2022). Introducing fairness considerations adds another layer of complexity, making the training process even more demanding. Therefore, model-centric approaches that attempt to enforce fairness during training may not be practical, as they introduce additional objectives that further complicate the already challenging training process (Liu, 2023).

To address this challenge, we propose FairLink, a data-centric approach that incorporates dyadic fairness regularizer into the learning of the enhanced graph. This is achieved by optimizing a fairness loss function jointly with a utility loss. The utility loss is computed by evaluating the gradient distance (Zhao et al., 2020; Jin et al., 2023, 2022a), which measures the differences in gradients between the enhanced and original graphs. This approach ensures that the task-specific performance is maintained in the learned graph (Zhao et al., 2020). Additionally, the dyadic fairness loss directs the learning process toward generating a *fair* graph for link prediction, while the utility loss ensures the preservation of link prediction performance. In contrast to model-centric approaches (Zha et al., 2023a,b; Jin et al., 2022b), which focus on designing fairness-aware link predictors, FairLinkemphasizes the creation of a generalizable fair graph specifically for link prediction tasks. We summarize our contributions as follows:

- This paper addresses the challenge of fair link prediction. While most existing methods concentrate on developing fairness-aware link predictors, we propose a novel data-centric approach. Our method focuses on constructing a fairness-enhanced graph. This graph can subsequently be used to train a link predictor without the need for debiasing techniques, while still ensuring fair link prediction.
- To ensure fairness in the fairness-enhanced graph, FairLink optimizes a dyadic fairness loss function. Additionally, to preserve utility, FairLink minimizes the gradient distance between the fairness-enhanced graph and the original input graph. To improve the measurement of gradient distance, we introduce a novel scale-sensitive distance function.
- The extensive experiments validate that, (1) the link prediction on the enhanced graph generated from FairLink is comparable with the link prediction on the input graph, (2) the fairness-utility trade-off of the enhanced graph is better than the baselines trained on the input graph, (3) the enhanced graph demonstrates strong generalizability, meaning it can achieve good fairness and utility performance on a test GNN architecture, even when it has been trained on a different GNN architecture.

## 2 Preliminaries

In the following section, we will start by introducing the notations used in our study. Next, we will explore the concept of fairness within the context of link prediction, which involves estimating the probability of a connection between two nodes in a network. We will then extend the principles of fair machine learning to the fairness of link prediction.

### 2.1 Notation

Let G=(V,E,X) as a graph, where V is the set of *N* nodes, E⊆V×V is the edge set, *X*∈ℝ^*N*×*D*^ is the node features with *D* dimensions. *A*∈{0, 1}^*N*×*N*^ is the adjacency matrix, where *A*_*uv*_ = 1 if there is an edge between nodes *u* and *v*. (*u, v*) denotes an edge between node *u* and node *v*. *S*∈ℝ^*N*×*K*^ is the vector containing sensitive attributes, *K* is the number of sensitive attributes can take on, (e.g., *S*_*u*_∈{Female, Male, Unkown} for node *u*). *g*(·, ·):ℝ^*H*^×ℝ^*H*^ → ℝ is the bivariate link predictor, and *g*(*z*_*u*_, *z*_*v*_) is the predicted probability of an edge (u,v)∈E in a given graph, where *z*_*u*_ and *z*_*v*_ are the node embedding vectors with dimension *H* for node *u* and *v*. The *problem* of fair link prediction aims to learn a synthetic graph $G_{f}=\left(V_{f},E_{f},X_{f}\right)$, where a link predictor *g*(·, ·) trained on $G_{f}$ will obtain comparable performance with it trained on the original graph G, and the link predictions are fair. In our experiemnts, $\left|V_{f}|=|V\right|$ and $X_{f}\in {\mathbb{R}}^{N\times D}$.

### 2.2 Fairness in link prediction

Previous research in fair machine learning has typically defined fairness in the context of binary classification as the condition where the predicted label is independent of the sensitive attribute. In the domain of link prediction, which involves estimating the probability of a link between pairs of nodes in a graph, fairness can be extended by ensuring that the estimated probability is independent of the sensitive attributes of the two nodes involved. In this subsection, we introduce two fairness concepts relevant to link prediction: demographic parity and equal opportunity.

#### 2.2.1 Demographic parity

Demographic Parity (DP) requires that predictions are independent of the sensitive attribute. It has been extensively applied in previous fair machine learning studies, and by replacing the classification probability with link prediction probability. In the context of link prediction, DP fairness requires that the predicted probability of a link's existence be independent of the sensitive attributes of both nodes in the link. This concept is also referred to as *dyadic fairness* in prior literature (Li et al., 2021), and is defined as follows:


(1) $$\begin{array}{l} P\left(g\left(u,v\right)|S_{u}=S_{v}\right)=P\left(g\left(u,v\right)|S_{u}\neq S_{v}\right). \end{array}$$

Ideally, achieving dyadic fairness entails predicting intra- and inter-link relationships at the same rate from a set of candidate links. The metric used to assess dyadic fairness in link prediction is as follows:


(2) $$\begin{array}{l} \Delta_{DP}=|P\left(g\left(u,v\right)|S_{u}=S_{v}\right)-P\left(g\left(u,v\right)|S_{u}\neq S_{v}\right)|. \end{array}$$

#### 2.2.2 Equal opportunity

Compared to Demographic Parity, which requires the probability of an instance being classified as a positive outcome to be equal for both sensitive groups, Equal Opportunity (EO) requires that, *among instances from the positive class*, the probability of being assigned a positive outcome is equal for both sensitive groups. In other words, EO ensures that the true positive rate is independent of the sensitive attribute. In link prediction, EO fairness requires that the probability of a link existing between two nodes be the same for node pairs within the same sensitive group as well as for node pairs from different sensitive groups. The formal definition of EO in link prediction is as follows:


(3) $$\begin{array}{l} P\left(g\left(u,v\right)|S_{u}=S_{v}\right)=P\left(g\left(u,v\right)|S_{u}\neq S_{v},\left(u,v\right)\in E\right). \end{array}$$

Specifically, for link prediction, EO requires that the predicted probability *g*(*u, v*) of an existing link (u,v)∈E should be equal for node pairs within the same sensitive group (*S*_*u*_ = *S*_*v*_) and for node pairs from different sensitive groups (*S*_*u*_≠*S*_*v*_). This approach aims to prevent any group from being unfairly disadvantaged. The method for assessing distance of EO fairness in link prediction is defined as follows:


(4) $$\begin{array}{l} \Delta_{EO}=|P\left(g\left(u,v\right)|S_{u}=S_{v}\right)-P\left(g\left(u,v\right)|S_{u}\neq S_{v}\right),\left(u,v\right)\in E|. \end{array}$$

## 3 Fairness-enhanced graph learning

**Figure 1:**
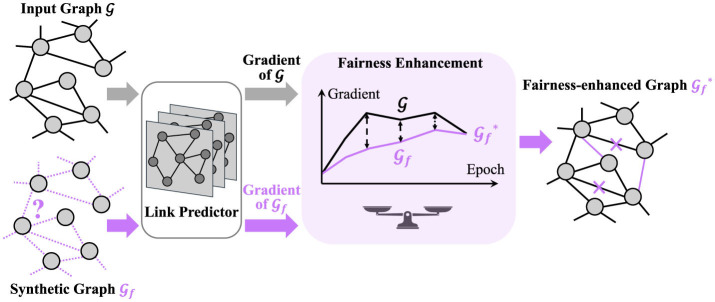
The overall framework of FairLink aims to learn a fairness-enhanced graph in which both fairness is promoted and utility is preserved. Initially, a synthetic graph $G_{f}$ is created with the same size as the input graph G and random link connections. Both the input graph and the synthetic graph are then fed into a trainable link predictor. The gradient of the cross-entropy loss with respect to the predictor's parameters is computed for both G and $G_{f}$. The optimization of $G_{f}$ involves minimizing a fairness loss in conjunction with the gradient distance between G and $G_{f}$.

In this section, we provide a comprehensive description of FairLink. Our objectives are twofold: (1) ensuring fairness within the fairness-enhanced graph and (2) preserving the utility of the fairness-enhanced graph. Specifically, our approach involves constructing a fairness-enhanced graph from the input graph to improve fairness in link prediction. To achieve the first objective, FairLink incorporates a dyadic regularization term that promotes fairness. For the second objective, FairLink maintains utility by minimizing the gradient distance between the input graph and the enhanced graph. Additionally, we introduce a novel scale-sensitive distance function to optimize the learned graph and measure the gradient distance effectively. To simplify the notation, we omit the training epoch *t* when introducing the loss function at a specific epoch. A framework of FairLink is provided in Figure 1.

### 3.1 Fairness enhancement

**Figure 2:**
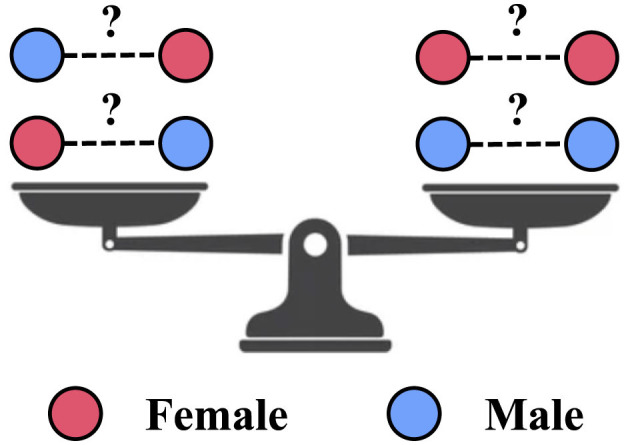
Fair link prediction objective in FairLink: Ensure equal probability for links between nodes from different sensitive groups and those from the same group.

In this subsection, we describe how to equip the learned graph with fairness-aware properties. This is achieved by incorporating a dyadic fairness regularizer, as specified in Equation 1, into the learning process of the fairness-enhanced graph. Further details on this process can be found in Section 2.2. The schematic diagram in Figure 2 illustrates the fairness objective of the fairness-enhanced graph learning within FairLink.

The concept of fairness constraint has been investigated in Zafar et al. (2015, 2017) by minimizing the disparity in fairness between users' sensitive attributes and the signed distance from the users' features to the decision boundary in fair linear classifiers. In this paper, we incorporate a fairness regularizer derived from Δ_*DP*_ (Chuang and Mroueh, 2021; Zemel et al., 2013), which quantifies the difference in the average predictive probability between various demographic groups. The fairness loss function $L_{fair}$ at training epoch *t* is defined as follows:


(5) $$\begin{array}{l} \begin{array}{c} L_{fair}=|{𝔼}_{u,v\sim V\times V}\left[g\left(u,v\right)|s_{u}=s_{v}\right]-{𝔼}_{u,v\sim V\times V}\left[g\left(u,v\right)|s_{u}\neq s_{v}\right]|, \end{array} \end{array}$$

where *E* is estimated $E_{u,v\sim V\times V}$ is generated from a link between any node pair in the graph. where $\hat{\text{Y}}$ represents the prediction probability of the downstream task. The variable *N* denotes the total number of instances, while *N*_**s** = 0/1_ refers to the total number of samples in the group associated with the sensitive attribute values of 0/1 respectively. The fundamental requirement for Δ_*DP*_ is that the average predictive probability $\hat{\text{Y}}$ within the same sensitive attribute group serves as a reliable approximation of the true conditional probability $\text{P}\left(\hat{\text{Y}}=1|\text{S}=0\right)$ or $\text{P}\left(\hat{\text{Y}}=1|\text{S}=1\right)$.

### 3.2 Utility preserving

In this section, we address the *first objective*: determining how to learn a fairness-enhanced graph such that a link predictor trained on it exhibits comparable performance to one trained on the input graph. FairLink first computes the link prediction loss for the original graph, denoted as L(G), by calculating the cross-entropy loss between the predicted link distribution (based on the dot product scores of the node embeddings) and the actual link distribution. Similarly, the link prediction loss for the synthetic graph, $L\left(G_{f}\right)$, is computed in the same manner. The gradients of both graphs with respect to the link predictors' weights, denoted as $\nabla_{\theta }L\left(G\right)$ and $\nabla_{\theta }L\left(G_{f}\right)$, are then obtained. We define the utility loss $L_{util}$ as the sum of the distances between these gradients across all training epochs.

Previous studies have utilized Cosine Distance to measure the distance between two gradients (Zhao et al., 2020; Jin et al., 2023; Liu and Shen, 2024b,a). While effective, Cosine Distance is scale-insensitive, meaning it ignores the magnitude of the vectors. Since the magnitude of the gradient is critical for optimization, incorporating it into the distance measurement is important. To address this limitation, we propose a combined approach that integrates Cosine Distance with Euclidean Distance, which accounts for vector magnitudes. Thus, the revised distance function *D* is defined as:


(6) $$\begin{array}{l} D\left(\nabla_{\theta_{t}}L\left(G\right),\nabla_{\theta_{t}}L\left(G_{f}\right)\right)=D_{cos}+\gamma D_{euc}, \end{array}$$

where *D*_cos_ denotes the Cosine Distance, *D*_euc_ denotes the Euclidean Distance, γ serves as a trade-off hyperparameter, and θ_*t*_ is the trainable parameters for link predictor at training epoch *t*. The definitions of these distances are as follows:


(7) $$\begin{array}{l} D_{cos}\left(\nabla_{\theta_{t}}ℒ(G),\nabla_{\theta_{t}}ℒ(G_{f})\right)=\sum_{i}\left(1-\frac{\nabla_{\theta_{t}}ℒ{\left(G\right)}_{i}\cdot \nabla_{\theta_{t}}ℒ{\left(G_{f}\right)}_{i}}{\left\|\nabla_{\theta_{t}}ℒ{\left(G\right)}_{i}\right\|\left\|\nabla_{\theta_{t}}ℒ{\left(G_{f}\right)}_{i}\right\|}\right),\\ D_{euc}\left(\nabla_{\theta_{t}}ℒ(G),\nabla_{\theta_{t}}ℒ(G_{f})\right)=\left\|\nabla_{\theta_{t}}ℒ{\left(G\right)}_{i}-\nabla_{\theta_{t}}ℒ{\left(G_{f}\right)}_{i}\right\|. \end{array}$$

The utility loss at a specific epoch *t*, denoted as $L_{\text{util}}$, is computed by summing the gradient distances between G and $G_{f}$ across all training epochs. It is formally defined as follows:


$$L_{util}=D\left(\nabla_{\theta_{t}}L\left(G\right),\nabla_{\theta_{t}}L\left(G_{f}\right)\right).$$

Minimizing the utility loss ensures that the training trajectory of $G_{f}$ closely follows that of G, leading to parameters learned on $G_{f}$ closely approximating those learned on G. As a result, $G_{f}$ preserves the essential information of the input graph G.

### 3.3 Optimization

Optimizing a fairness-enhanced graph directly is computationally expensive due to the quadratic complexity involved in learning **A**_*f*_. To address this challenge, previous work (Jin et al., 2023) proposed modeling **A**_*f*_ as a function of **X**_*f*_. We initialize the node feature **X**_*f*_ by randomly selecting original features from each class. Note that learning a fairness-aware feature matrix for fair link prediction is important because this matrix will be used for node embedding when training a new link predictor on the fairness-enhanced graph. Therefore, it is necessary for the feature matrix itself to be fairness-aware. We further simplify this approach by using a multi-layer perceptron parameterized by ψ with a sigmoid activation function to model the relationship, thereby reducing the computational burden. Thus, the final loss function is as follows:


(8) $$\begin{array}{l} \underset{{\text{X}}_{f},\psi }{\min}{𝔼}_{\theta_{0}\sim P_{\theta_{0}}}\left[\overset{T-1}{\underset{t=0}{\sum }}\left(L_{util}+\alpha L_{fair}+\beta ||\theta_{t}|{|}^{2}\right)\right], \end{array}$$

where *T* is the total training epochs, α and β are hyperparameters that govern the influence of two critical aspects: the gradient matching loss and the *L*_2_ norm regularization, respectively.

Jointly optimizing **X**_*f*_ and ψ is often challenging due to the interdependence between them. To overcome this, we employ an alternating optimization strategy. We first update **X**_*f*_ for τ_1_ epochs, then update ψ for τ_2_ epochs. This process is repeated alternately until the stopping criterion is satisfied.

### 3.4 Fair link prediction

To achieve fair link prediction, we first use the fairness-enhanced graph $G_{f}$ to train a link predictor. This link predictor can differ in architecture from the model that produced $G_{f}$ and does not necessarily incorporate fairness considerations. In this paper, we define the link prediction function *g*(·, ·) as the inner product between the embeddings of two nodes *u* and *v*, for each node pair (u,v)∈V×V. Specifically, the function is defined as *g*(*u, v*) = *u*^⊤^Σ*v*, where Σ is a positive-definite matrix that scales the input vectors directionally. In our implementation, Σ is set to an identity matrix, simplifying *g*(·, ·) to the dot product, which is commonly used in link prediction research (Trouillon et al., 2016; Kipf and Welling, 2016b).

## 4 Discussion

In this paper, the fairness-enhanced graph produced by FairLink retains the same size as the input graph, as discussed in Section 2.1. To facilitate fairness-aware training on large-scale graphs, our approach concentrates on learning a fairness-enhanced graph that can be reused, thereby eliminating the need for repeated debiasing in future training with different link predictors. Future work could investigate methods for learning a smaller, fairness-enhanced graph derived from large-scale real-world graphs.

## 5 Experiments

In this section, we evaluate the effectiveness of FairLink on four large-scale real-world graphs. We focus on assessing its performance in link prediction and fairness, as well as the trade-off between fairness and utility by comparing FairLink with seven baseline methods. Additionally, we examine the generalizability of the graphs generated by FairLink by applying them to various GNN architectures.

### 5.1 Experimental setup

#### 5.1.1 Datasets

We consider four large-scale graphs that have been extensively used in previous studies on fair link prediction. These graphs span a diverse range of domains, including citation networks, co-authorship networks, and social networks, each characterized by different sensitive attributes. We consider the nodes that take minority as the protected node group (e.g., Female nodes Google+ and male nodes in the Facebook). The statistics of the datasets are in Table 1.

- **Pubmed**^1^: Pubmed is a dataset where each node represents an article, characterized by a bag-of-words feature vector. An edge between two nodes indicates a citation between the corresponding articles, regardless of direction. The topic of an article, i.e., its category, is used as the sensitive attribute in this dataset.
- **DBLP**^2^ (Tang et al., 2008): DBLP is a co-authorship network constructed from the DBLP computer science bibliography database. The network comprises nodes representing authors extracted from papers accepted at eight different conferences. An edge exists between two nodes if the corresponding authors have collaborated on at least one paper. The sensitive attribute in this dataset is the continent of the author's institution.
- **Google+**^3^ (Leskovec and Mcauley, 2012): Google+ is a social network dataset. The data was collected from users who chose to share their social circles, where they manually categorized their friends on the Google+ platform.
- **Facebook**^4^ (Leskovec and Mcauley, 2012): Facebook is a dataset that contains anonymized feature vectors for each node, representing various attributes of a person's Facebook profile.

**Table 1:** Link prediction and fairness results on large-scale graphs.

**Metric**	**VGAE**	**Node2vec**	**FairPR**	**Fairwalk**	**FairAdj**	**FLIP**	**FairEGM**	**FairLink**
**Pubmed** **#Nodes:** 19, 717 **#Edges:** 88, 648 **Sensitive Attribute: Topic**								
F1 (↑)	**93.18**±1.07	86.50 ± 1.48	83.33 ± 2.79	85.20 ± 2.53	84.25 ± 1.21	83.48 ± 1.79	83.70 ± 1.68	90.46±1.67
AUC (↑)	**96.20**±0.85	93.27 ± 1.23	88.21 ± 0.62	91.43 ± 1.11	90.53 ± 1.03	87.44 ± 1.36	88.12 ± 2.33	95.24±1.65
Δ_*DP*_ (↓)	20.88 ± 12.48	19.14 ± 11.93	17.31 ± 6.32	18.42 ± 8.65	14.73±5.98	15.42 ± 7.69	17.52 ± 6.30	**5.42**±2.65
Δ_*EO*_ (↓)	18.84 ± 10.98	20.33 ± 8.74	15.39 ± 9.52	20.18 ± 7.75	16.39±4.64	19.43 ± 8.01	19.29 ± 9.44	**4.86**±1.34
**DBLP** **#Nodes:** 13, 015 **#Edges:** 79, 972 **Sensitive Attribute: Continent**								
F1 (↑)	**82.23**±1.66	78.15 ± 1.72	80.05 ± 1.27	80.88 ± 2.81	81.62 ± 1.58	77.62 ± 1.71	80.45 ± 0.92	81.69±1.55
AUC (↑)	**90.77**±1.82	83.21 ± 2.94	72.43 ± 1.30	88.39 ± 1.59	84.51 ± 2.25	78.14 ± 3.41	80.43 ± 2.62	88.72±1.76
Δ_*DP*_ (↓)	7.42 ± 3.95	8.43 ± 5.25	11.65 ± 4.33	9.86 ± 4.04	3.55±3.37	6.34 ± 4.22	5.82 ± 5.33	**1.32**±0.45
Δ_*EO*_ (↓)	8.53 ± 3.60	7.22 ± 4.37	9.37 ± 5.24	7.10 ± 3.57	5.82 ± 3.91	5.39±4.37	7.33 ± 6.32	**2.19**±1.01
**Google+** **#Nodes:** 4, 938 **#Edges:** 547, 923 **Sensitive Attribute: Gender**								
F1 (↑)	**88.33**±1.21	81.11 ± 1.50	76.22 ± 1.36	82.47 ± 1.08	84.77 ± 1.19	78.35 ± 2.02	80.69 ± 1.53	85.34±0.81
AUC (↑)	**94.85**±0.91	88.74 ± 2.84	67.29 ± 1.53	93.01 ± 0.58	93.37 ± 0.22	81.86 ± 1.54	80.26 ± 1.61	94.42±1.86
Δ_*DP*_ (↓)	6.42 ± 2.05	7.88 ± 4.72	7.14 ± 1.83	5.61 ± 4.20	3.79 ± 1.22	**1.19**±1.93	4.55 ± 2.11	1.42±0.96
Δ_*EO*_ (↓)	7.92 ± 4.48	9.35 ± 3.19	6.35 ± 3.09	4.42 ± 1.93	3.76 ± 1.47	2.21±1.12	5.37 ± 3.65	**1.01**±0.75
**Facebook** **#Nodes:** 1, 045 **#Edges:** 53, 498 **Sensitive Attribute: Gender**								
F1 (↑)	**82.41**±1.23	79.35 ± 0.95	76.22 ± 1.30	78.11 ± 0.78	81.14 ± 1.23	78.5 ± 1.42	79.77 ± 2.92	82.37±0.41
AUC (↑)	**94.66**±0.55	90.57 ± 1.24	70.30 ± 1.09	91.56 ± 0.63	92.53 ± 1.49	83.0 ± 1.51	85.42 ± 1.45	93.73±1.72
Δ_*DP*_ (↓)	2.03 ± 0.81	1.70 ± 1.43	2.33 ± 1.91	1.97 ± 1.51	1.77 ± 0.81	1.17±0.55	2.21 ± 1.05	**0.83**±0.36
Δ_*EO*_ (↓)	3.78 ± 2.15	2.10 ± 1.60	2.95 ± 1.10	1.83 ± 1.39	**1.25**±0.74	2.21 ± 1.52	2.55 ± 1.34	1.56±2.21

An upward arrow (↑) indicates that a higher value is better, while a downward arrow (↓) signifies the opposite. For each metric, the best results are highlighted in bold, and the runner-up results are underlined.

## 6 Baselines

We compare with two link prediction approaches, VGAE and Node2vec, and five state-of-the-art fair link prediction methods, FairPR, Fairwalk, FairAdj, FLIP, and FairEGM.

- **Link prediction methods**: We consider two popular link prediction baselines: (1) The Variational Graph Autoencoder (VGAE) (Kipf and Welling, 2016b), which is based on the variational autoencoder model. VGAEuses a GNN as the inference model and employs latent variables to reconstruct graph connections. (2) Node2vec (Grover and Leskovec, 2016), a widely-used graph embedding approach based on random walks. It represents nodes as low-dimensional vectors that capture proximity information, enabling link prediction through these node embeddings.
- **Fair link prediction methods**: To evaluate fairness in link prediction, we compare against five state-of-the-art approaches: (1) FairPR (Tsioutsiouliklis et al., 2021), which extends the PageRank algorithm by incorporating group fairness considerations. (2) Fairwalk (Rahman et al., 2019), built upon Node2vec, modifies transition probabilities during random walks based on the sensitive attributes of a node's neighbors to promote fairness. (3) FairAdj (Li et al., 2021), a regularization-based link prediction algorithm, ensures dyadic fairness by maintaining the utility of link prediction through a VGAE, while enforcing fairness with a dyadic loss regularizer. (4) FLIP (Masrour et al., 2020) enhances structural fairness in graphs by reducing homophily and evaluates fairness by measuring reductions in modularity. (5) FairEGM (Current et al., 2022), a collection of three methods, emulates different types of graph modifications to improve fairness. In our experiments, we use Constrained Fairness Optimization (GFO) as the representative method from this collection.

For a detailed discussion of the fair link prediction baselines, please refer to Section 10.3.

## 7 Metrics

To evaluate the accuracy of link prediction, we use two metrics: the F1-score and the area under the receiver operating characteristic curve (AUC) (Current et al., 2022; Li et al., 2021; Masrour et al., 2020). For assessing group fairness, we adopt two additional metrics: the difference in demographic parity (Δ_*DP*_) (Feldman et al., 2015) and the difference in equal opportunity (Δ_*EO*_) (Hardt et al., 2016), as introduced in Section 2.2. Lower values of Δ_*DP*_ and Δ_*EO*_ indicate better fairness, making these metrics crucial for evaluating the fairness of the model.

## 8 Protocols

For the implementation of FairLink, we utilize a two-layer GraphSAGE (Hamilton et al., 2017) as the feature embedding and inference mechanism. For VGAE and Node2vec, we adhere to the hyperparameter settings outlined in Masrour et al. (2020), while for the other baselines, we follow the configurations provided in their respective original papers. To fine-tune the model, we perform a grid search over the hyperparameters α, β, and γ for each dataset. Specifically, α and β are selected from the set {0.001, 0.005, 0.01, 0.05, 0.1, 0.5, 1.0, 1.5, 2, and 2.5}, and γ is chosen from {0.2, 0.4, 0.6, 0.8, 1.0, 1.2, 1.4, 1.6, 1.8, 2.0, and 2.5}. Each experiment is conducted 10 times, with training set to 200 epochs across all datasets. The learning rate is specifically tuned for each dataset: 0.005 for Pubmed, and 0.01 for DBLP, Google+, and Facebook. All losses are optimized using the Adam optimizer (Kingma and Ba, 2014). We followed the splitting from previous studies (Gurukar et al., 2019; Current et al., 2022), and conducted all experiments across 10 runs, employing different random seeds and train/test splits for each run.

### 8.1 Link prediction and fairness performance of FairLink

To evaluate the performance of our proposed method in both link prediction and fairness, we conducted a comprehensive comparison with the previously mentioned baselines using four real-world datasets. The results, which include the mean and standard deviations for all models across these datasets, are detailed in Table 1. From these results, we can draw the following observations:

- Our proposed method, FairLink, consistently demonstrates superior fairness performance in terms of both Δ_*DP*_ and Δ_*EO*_ across all evaluated datasets. For example, compared to VGAE, FairLinkreduces Δ_*DP*_ by 74.0%, 82.2%, 77.9%, and 59.1% on the Pubmed, DBLP, Google+, and Facebook, respectively.
- Regarding utility, FairLinktypically achieves the second-best performance in terms of F1-score and AUC. For instance, FairLinkretains 97.08%, 99.26%, 96.61%, and 99.95% of the F1-score of VGAEon the Pubmed, DBLP, Google+, and Facebookdatasets, respectively.
- Fair link prediction baselines, such as FairPR, Fairwalk, FairAdj, FLIP, and FairEGM, exhibit less predictive bias compared to standard link prediction models like VGAE and Node2vec. Among these, fairadj generally performs second-best after FairLink. Specifically, FairAdj shows better performance on the Facebook, while FLIP outperforms the others on the Google+.

### 8.2 Fairness-utility trade-off comparison

**Figure 3:**
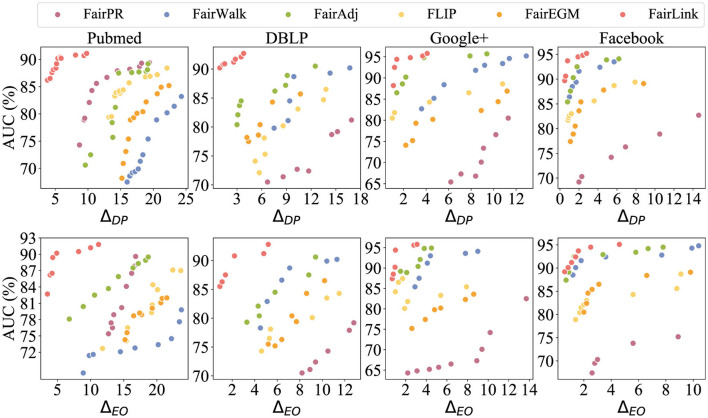
Trade-off between fairness and link prediction accuracy across four datasets. Results in the upper left corner, which exhibit both lower bias and higher accuracy, represent the ideal balance.

In Figure 3, different colors are employed to distinguish between FairPR, Fairwalk, FairAdj, FLIP, FairEGM, and FairLink. Ideally, a debiasing method should be positioned in the upper-left corner of the plot to achieve the optimal balance between utility and unbiasedness. As depicted in the figures, methods based on FairLink generally provide the most favorable trade-offs between these two competing objectives. In contrast, while FairAdj usually offers superior debiasing with minimal utility loss, Fairwalk excels in maintaining high utility but is less effective in reducing bias. Although FairPR can achieve reasonable unbiasedness in embeddings, it significantly compromises utility compared to FairLink, as illustrated in the DBLP and Google+ datasets. In contrast, FairEGM does not show a notable debiasing effect.

The ability of FairLink to achieve a superior trade-off is attributed to its objective design, as outlined in Equation 8. Previous studies have demonstrated the effectiveness of gradient matching in generating synthetic data that maintains prediction accuracy during machine learning model training (Zhao et al., 2020; Jin et al., 2023, 2022a; Liu and Shen, 2024a). It is important to note that the synthetic graph derived from minimizing the gradient matching objective is not a singular solution; rather, it is quite flexible. Inspired by this insight, the proposed FairLink seeks to promote fairness in the learned data–the fairness-enhanced graph–by incorporating a fairness constraint into the gradient matching objective. In essence, FairLink aims to identify a solution that is close to the optimized graph space, where the gradient matching loss is zero, while simultaneously minimizing the fairness loss. A prior study also confirms the favorable fairness-utility trade-off in the learned graph when utilizing this design for the node classification task (Liu, 2023).

### 8.3 Generalizability to other link prediction models

**Figure 4:**
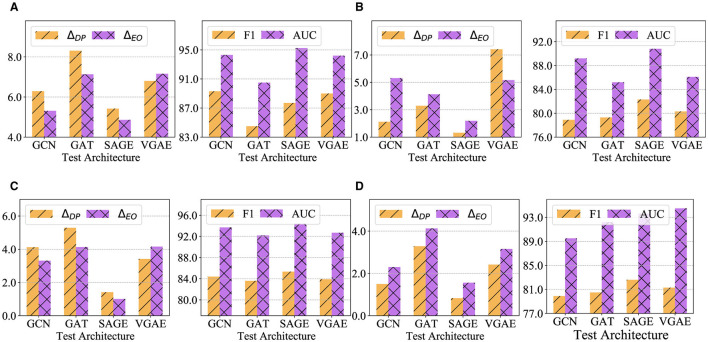
Cross-architecture performance of FairLinkwith different test architectures on four datasets. **(A)** Pubmed. **(B)** DBLP. **(C)** Google+. **(D)** Facebook.

To validate the generalizability of the fairness-enhanced graph, we perform a cross-architecture analysis. Initially, we used GraphSAGE (SAGE) to generate synthetic graphs. These graphs are then evaluated across various architectures, including GCN, GAT, and VGAE, as well as on the original GraphSAGE model. Additionally, we apply FairLink with different structures to all datasets and assess the cross-architecture generalization performance of the fairness-enhanced graphs. The results of these experiments are documented in Figures 4A–D.

Compared to Table 1, FairLinkdemonstrates improved fairness performance over VGAEand Node2vecacross most model-dataset combinations. This indicates that FairLinkis versatile and consistently achieves gains across various architectures and datasets. Our fairness-enhanced graphs show generally superior performance in fairness metrics (e.g., Δ_*DP*_ and Δ_*EO*_) and utility metrics (e.g., F1-score and AUC) across all datasets. Specifically, GraphSAGE excels in fairness across all datasets and achieves the best utility on Pubmed, DBLP, and Google+.

### 8.4 Ablation study

To evaluate the necessity of generating a synthetic graph for fair link prediction in architectures without built-in fairness considerations, we conducted an ablation study to compare the performance of the proposed FairLink with two of its variants: (1) FairLink*m*, which is a model-centric variant of FairLink, excludes the synthetic graph and directly applies the dyadic fairness loss in Equation 5 to the training of a link predictor, and (2) FairLink*cos*, which only uses the cosine distance function in the gradient matching process in Equation 6, by setting γ = 0. We evaluated both link prediction accuracy and fairness performance across various architectures that do not explicitly account for fairness.

For FairLink, we first trained GraphSAGE to obtain a fairness-enhanced graph. This graph was then used for training diverse architectures without any fairness design, including GraphSAGE, GAT, and VGAE, and we tested on the trained link predictor. In the variant without the synthetic graph generation, we incorporated the fairness loss directly into the training of GraphSAGE. However, similar to FairLink, we excluded fairness loss when training the other architectures, such as GAT and VGAE, to ensure a fair comparison.

**(1) Data-centric vs. model-centric debiasing:** From the results in the “(GAT)” and “(VGAE)” columns of Table 2, which correspond to architectures without fairness-aware designs, we observe that FairLinkcan generalize fairness when applied to different architectures, whereas FairLink*m* cannot. Comparing the “(Self)” columns for FairLink and FairLink*m*, it is evident that directly adding fairness loss during the training of a link predictor significantly degrades accuracy. This aligns with findings from prior studies, where applying fairness loss directly during the training of node classifiers led to a similar drop in performance (Qian et al., 2024; Dong et al., 2024). However, FairLink, by utilizing a gradient matching loss to preserve link prediction accuracy on the fairness-enhanced graph, successfully alleviates this trade-off. As a result, FairLink achieves both higher accuracy and better generalization of fairness across different architectures.

**Table 2:** An ablation study comparing the proposed FairLink with its variants FairLink*m* and FairLink*cos*.

**Method**	**Metric**	**DBLP(Self)**	**DBLP(GAT)**	**DBLP(VGAE)**	**Google+(Self)**	**Facebook(GAT)**	**Google+(VGAE)**
FairLink	F1 (↑)	81.69	79.31	80.28	85.34	83.62	83.94
	AUC (↑)	88.72	85.22	86.19	94.42	92.20	92.65
	Δ_*DP*_ (↓)	1.32	3.29	7.42	1.42	5.29	3.42
	Δ_*EO*_ (↓)	2.19	4.13	5.16	1.01	4.13	4.16
FairLink *m*	F1 (↑)	78.57	79.90	80.35	83.80	80.04	83.23
	AUC (↑)	84.32	84.34	86.36	91.05	89.36	91.90
	Δ_*DP*_ (↓)	4.25	7.27	11.31	3.83	7.72	8.71
	Δ_*EO*_ (↓)	5.73	8.14	9.75	3.92	8.10	9.22
FairLink *cos*	F1 (↑)	79.15	78.63	78.06	81.17	79.84	80.85
	AUC (↑)	86.24	82.25	85.32	89.23	87.48	88.45
	Δ_*DP*_ (↓)	1.41	5.19	8.11	1.31	5.97	4.37
	Δ_*EO*_ (↓)	2.76	4.78	5.73	1.25	5.68	3.28

The columns labeled “(Self)” indicate that both the training and testing architectures are GraphSAGE, with fairness constraints applied during training. In contrast, the columns labeled “(GAT)” or “(VGAE)” indicate that the test architectures are GAT or VGAE, respectively, and do not incorporate fairness-aware design. An upward arrow (↑) indicates that a higher value is better, while a downward arrow (↓) signifies the opposite.

**(2) Euclidean & cosine distance vs. cosine distance:** From the results of FairLink and FairLink*cos*, we observe a decline in link prediction performance when the Euclidean function is excluded from gradient matching. This finding highlights the importance of minimizing the gradient magnitude when optimizing the fairness-enhanced graph. In conclusion, FairLink, which utilizes both Euclidean and Cosine distance functions, is more effective at preserving the original graph's information in the learned graph.

## 9 Further discussions

### 9.1 Complexity analysis

Let *L* denote the number of MLP layers used for learning the adjacency matrix, and let *d* represent the number of hidden units. The complexity of FairLink is constituted by several calculations: (1) Calculation of *A*′: This step has a complexity of *O*(*N*^2^*d*^2^). (2) Forward Pass of GNN: Computing the forward pass on the full graph incurs a complexity of *O*(*kLNd*^2^), where *k* is the size of the sampled nodes per training instance. (3) Training on Fairness-Enhanced Graph: The complexity for training on the fairness-enhanced graph is *O*(*LNd*). (4) Gradient Matching Strategy: Including the calculation of additional matching metrics, the complexity of the gradient matching strategy is *O*(2|θ|+|*A*′|+|*X*′|). Considering *T* iterations and *M* different initializations, the total complexity is *MT* times the sum of the aforementioned complexities. (5) For link prediction tasks, calculating the dot product of node embeddings adds an extra cost of *O*(*N*^2^*D*). Therefore, the overall complexity of FairLink is the sum of all these components.

### 9.2 A smaller size of the fairness-enhanced graph

To evaluate whether it is feasible to learn a smaller fairness-enhanced graph, we implement method by initializing a synthetic graph with 75% of the nodes from the input training graph. In this experiment, we fine-tune all the hyperparameters in FairLink using the same settings as for the full graph on the DBLPdataset. We then compare the performance of a link predictor trained on both the full fairness-enhanced graph and the smaller graph. We assess the performance on two different architectures: the same architecture used for generating the graph (labeled as “Self”) and a different architecture (labeled as “GAT”).

From the results presented in Table 3, we find that FairLink is capable of learning a compact and generalizable fairness-enhanced graph for DBLPdataset. This demonstrates the scalability of FairLink for large-scale graphs and highlights its potential to be applied in the wild.

**Table 3:** An ablation study comparing the proposed FairLink with its variant FairLink*m*.

**Metric**	**DBLP(Full, Self)**	**DBLP(Full, GAT)**	**DBLP(75*%*, Self)**	**DBLP(75*%*, GAT)**
F1 (↑)	81.69	79.31	80.67	78.52
AUC (↑)	88.72	85.22	88.02	83.29
Δ_*DP*_ (↓)	1.32	3.29	1.63	4.37
Δ_*EO*_ (↓)	2.19	4.13	2.75	5.33

The columns labeled “(Self)” indicate that both the training and testing architectures are GraphSAGE, with fairness constraints applied during training. In contrast, the columns labeled “(GAT)” or “(VGAE)” indicate that the test architectures are GAT or VGAE, respectively, and do not incorporate fairness-aware design. An upward arrow (↑) indicates that a higher value is better, while a downward arrow (↓) signifies the opposite.

### 9.3 Choice of FairLink architecture

To identify the optimal architecture for FairLink, we conduct a cross-architecture experiment using different graph generation models. Specifically, we use one architecture to generate the fairness-enhanced graph and another architecture to train on the generated graph, followed by performance evaluation through testing.

From the results in Table 4A, we can find that the highest link prediction accuracy is achieved when the same architecture is used for both generation and testing. While GraphSAGE and VGAE exhibit similar levels of accuracy, a key distinction emerges when examining generalization performance. Specifically, fairness-enhanced graphs generated by GraphSAGE demonstrate better generalizability across different architectures, such as GCN, GAT, and VGAE. Furthermore, although both GCN and GraphSAGE show comparable fairness performance as shown in Table 4B, GraphSAGE exhibits a slight advantage in terms of generalization.

**Table 4:** Cross-architecture experiment results on various generation and testing architectures.

**(A) DBLP, F1**				
**Gen**\**Te**	**GCN**	**GAT**	**SAGE**	**VGAE**
GCN	**79.3**	77.4	76.2	78.4
GAT	76.0	**79.5**	79.3	77.8
SAGE	78.9	79.3	**82.3**	80.3
VGAE	77.6	78.6	78.2	**81.8**
**(B) DBLP**, Δ_*DP*_				
**Gen**\**Te**	**GCN**	**GAT**	**SAGE**	**VGAE**
GCN	**1.21**	3.52	1.79	4.65
GAT	3.19	**2.40**	2.26	4.15
SAGE	2.12	3.29	**1.32**	3.87
VGAE	2.71	3.65	3.25	**3.09**

We report the F1 score and Δ_*DP*_ on the DBLPdataset. “SAGE” refers to GraphSAGE, “Gen” refers to generation, and “Te” represents testing. For each metric, the best results are highlighted in bold.

## 10 Related work

### 10.1 Fairness in machine learning

In recent years, numerous fairness definitions in machine learning have been proposed. These definitions generally fall into three categories. (1) *Group fairness*, which aims to ensure that certain statistical measures are approximately equal across protected groups (e.g., racial or gender groups) (Feldman et al., 2015; Hardt et al., 2016). (2) *Individual fairness* (Dwork et al., 2012; Kang et al., 2020; Dong et al., 2021, 2023) requires that similar individuals should be treated similarly. Compared with group fairness, individual fairness does not take sensitive features into account. Instead, it emphasizes fairness at the individual level, such as for each node in graph data. (3) *Counterfactual fairness* (Kusner et al., 2017; Ma et al., 2022; Zuo et al., 2022) seeks to ensure fairness by considering how decisions would hold under alternative scenarios. Counterfactual fairness is achieved when the prediction results for an individual remain consistent across their counterfactuals. In this context, “counterfactuals” refer to different versions of the same individual, where their sensitive features have been altered to various values. Thus, the prediction outcomes for the individual and their counterfactuals should be identical. In our experiments, we adopt two widely used definitions of group fairness: demographic parity and equal opportunity. Demographic parity (Feldman et al., 2015) requires that members of different protected classes are represented in the positive class at the same rate, meaning the distribution of protected attributes in the positive class should reflect the overall population distribution. In contrast, equal opportunity (Hardt et al., 2016) focuses on the model's performance rather than just the outcome; it requires that true positive rates are equal across different protected groups, ensuring that the model performs consistently for all groups. Methodologically, existing bias mitigation techniques in machine learning can be broadly categorized into three approaches: (1) *Pre-processing*, where bias is mitigated at the data level before training begins (Calders et al., 2009; Kamiran and Calders, 2009; Feldman et al., 2015); (2) *In-processing*, where the machine learning model itself is modified by incorporating fairness constraints during training (Zafar et al., 2017; Goh et al., 2016); and (3) *Post-processing*, where the outcomes of a trained model are adjusted to achieve fairness across different groups (Hardt et al., 2016).

### 10.2 Link prediction

Link prediction involves inferring new or previously unknown relationships within a network. It is a well-studied problem in network analysis, with various algorithms developed over the past two decades (Liben-Nowell and Kleinberg, 2003; Al Hasan et al., 2006; Hasan and Zaki, 2011). Specifically, *heuristic methods* define a score based on the graph structure to indicate the likelihood of a link's existence (Liben-Nowell and Kleinberg, 2003; Newman, 2001; Zhou et al., 2009). The primary advantage of heuristic methods is their simplicity, and most of these approaches do not require any training. *Graph embedding methods* learn low-dimensional node embeddings, which are then used to predict the likelihood of links between node pairs (Grover and Leskovec, 2016; Menon and Elkan, 2011). These embeddings are typically trained to capture the structural properties of the graph. *Deep neural network methods* have emerged as state-of-the-art for the link prediction task in recent years (Kipf and Welling, 2016a,b; Hamilton et al., 2017; Velickovic et al., 2018). This category includes GNNs, which leverage the multi-hop graph structure through the message-passing paradigm. Additionally, GNNs augmented with auxiliary information, such as pairwise information (Zhang M. et al., 2021), have been proposed to enhance link prediction. These advanced methods incorporate additional data to better capture the relationships between nodes (Zhang M. et al., 2021; Zhu et al., 2021; Wang et al., 2022).

### 10.3 Fair link prediction

With the success of GNNs, there has been increasing attention on fairness in graph representation learning (Dai et al., 2022). Some works have focused on creating fair node embeddings, which are subsequently used in link prediction (Bose and Hamilton, 2019; Buyl and De Bie, 2020; Cui et al., 2018). Others have directly targeted the task of fair link prediction (Masrour et al., 2020; Li et al., 2021). Specifically, *dyadic fairness* has been proposed for link prediction, which requires the prediction to be independent of whether the two vertices involved in a link share the same sensitive attribute (Li et al., 2021). To achieve dyadic fairness, the authors proposed FairAdj (Li et al., 2021), which leverages a variational graph auto-encoder (Kipf and Welling, 2016b) for learning the graph structure and incorporates a dyadic loss regularizer to enforce fairness. FairPageRank (FairPR) (Tsioutsiouliklis et al., 2021) is a fairness-sensitive variation of the PageRank algorithm. It modifies the jump vector to ensure fairness, both globally and locally. The locally fair PageRank variant specifically guarantees that each node behaves in a fair manner individually. *DeBayes* (Buyl and De Bie, 2020) adopts a Bayesian approach to model the structural properties of the graph, aiming to learn debiased embeddings using biased prior conditional network embeddings. Meanwhile, Fairwalk (Rahman et al., 2019) adapts Node2vec (Grover and Leskovec, 2016) to enhance fairness in node embeddings by adjusting the transition probabilities in random walks, weighing the neighborhood of each node based on their sensitive attributes. Finally, FLIP (Masrour et al., 2020) tackles graph structural debiasing by reducing homophily (the tendency of similar nodes to connect) in the graph. The fairness is assessed by the reduction in modularity, which measures the strength of the division of a graph into modules. FairEGM (Current et al., 2022), a collection of three methods that emulate the effects of a variety of graph modifications for the purpose of improving graph fairness.

## 11 Conclusion

We study fairness in link prediction. Existing methods primarily focus on integrating debiasing techniques during training to learn unbiased graph embeddings. However, these methods complicate the training process, especially when applied to large-scale graphs. Additionally, they are model-specific, requiring a redesign of the debiasing approach whenever the model changes. To address these challenges, we propose a data-centric debiasing method, FairLink, which aims to enhance fairness in link prediction without modifying the training of large-scale graphs. FairLink optimizes both fairness and utility by learning a fairness-enhanced graph. It minimizes the difference between the training trajectory of the fairness-enhanced graph and the input graph, incorporating fairness loss in the training of the fairness-enhanced graph. Extensive experiments on benchmark datasets demonstrate the effectiveness of FairLink, as well as its ability to generalize across different GNN architectures.

## Data Availability

The original contributions presented in the study are included in the article/supplementary material, further inquiries can be directed to the corresponding author.

## Footnotes

^1^Pubmed: https://linqs.org/datasets/.

^2^DBLP: https://dblp.dagstuhl.de/xml/.

^3^Google+: https://snap.stanford.edu/data/ego-Gplus.html.

^4^Facebook: https://snap.stanford.edu/data/ego-Facebook.html.
